# Hyperhomocysteinemia Causing Myocardial Infarction in a Young Patient: A Case Report

**DOI:** 10.7759/cureus.77421

**Published:** 2025-01-14

**Authors:** Mamoon Qadir, Sabina Aslam, Bushra lail Shah, Amna Akbar, Sarosh Khan Jadoon

**Affiliations:** 1 Cardiology, Federal Government Poly Clinic, Islamabad, PAK; 2 Emergency and Accident, District Headquarter Hospital Jhelum Valley, Muzaffarabad, PAK; 3 General Surgery, Combined Military Hospital, Muzaffarabad, PAK

**Keywords:** acute myocardial infarction, case report, homocysteine, hyperhomocysteinemia, myocardial infarction

## Abstract

Hyperhomocysteinemia is an independent risk factor for acute myocardial infarction (MI). This case report describes a 19-year-old male with a marfanoid phenotype and no conventional risk factors presenting with acute MI. It highlights the significance of acknowledging hyperhomocysteinemia as a potential risk factor for MI, especially in young patients.

## Introduction

An association between increased homocysteine levels and coronary heart disease has already been established [1]. Several case reports have been published on this condition [2-6]. All the cases included patients above 20 years of age. This case report identifies increased homocysteine levels as a cause of acute myocardial infarction (MI) in a patient of less than 20 years of age.

## Case presentation

**Figure 1:**
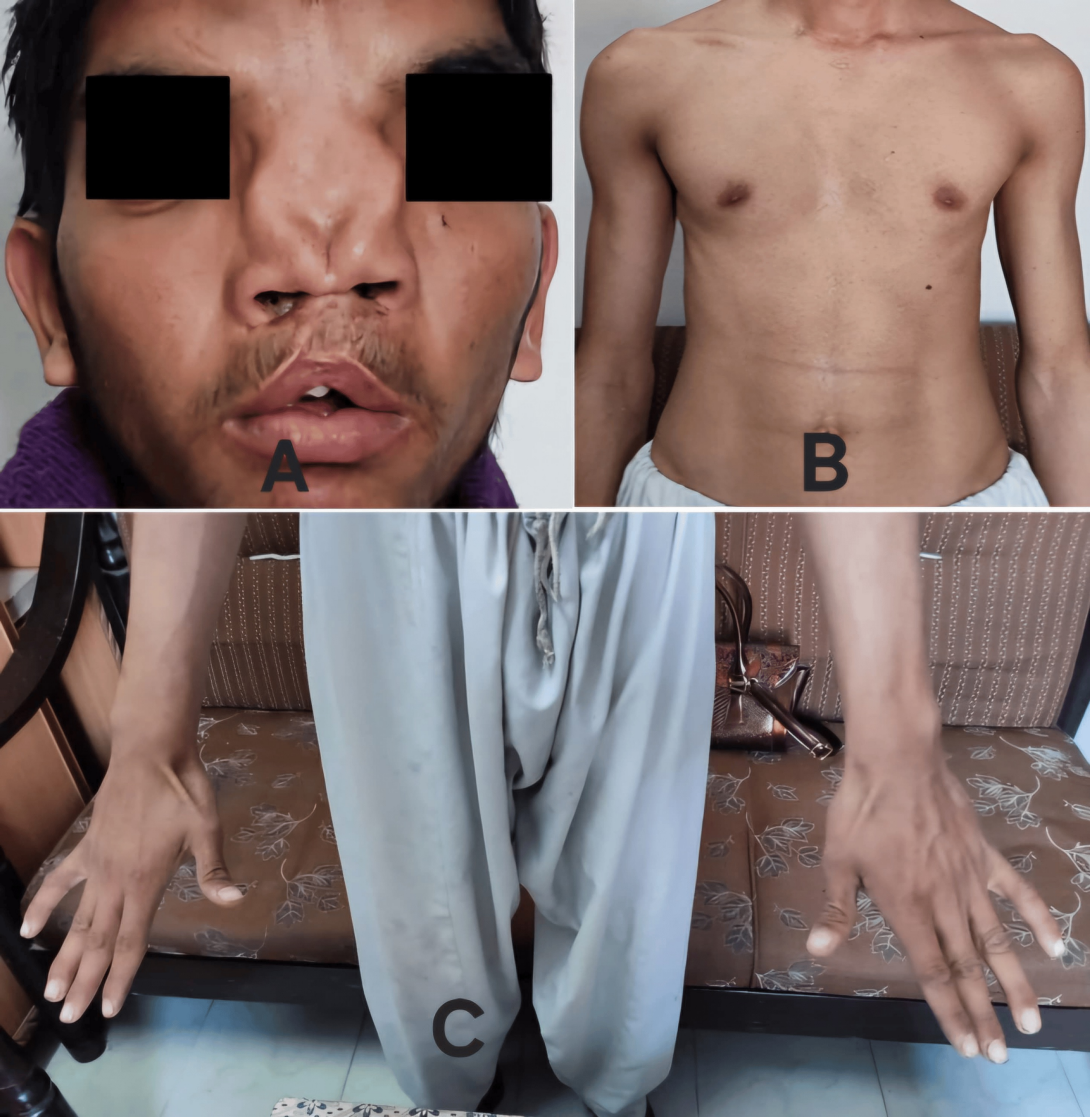
Patient characteristics Patient with depressed nasal bridge, cleft lip and high-arched palate (A), pectus excavatum (B), and disproportionately long hands (C).

A 19-year-old young man, with marfanoid phenotype, was brought to the emergency room of a tertiary care hospital in Pakistan complaining of central chest pain. The pain was crushing in nature, progressive, radiating into the back and left arm for six hours, accompanied by shortness of breath and apprehensions. At the time of presentation, the patient had a blood pressure of 100/75 mmHg, respiratory rate of 25 breaths/min, pulse rate of 63 beats/min, and temperature of 98-degree Fahrenheit. On general physical examination, he had a depressed nasal bridge, cleft lip, high-arched palate, pectus excavatum, and disproportionately long hands (Figure 1). Systemic examination was unremarkable.The patient consented to the disclosure of his identity in the form of images and the journal has received the signed consent form.

Past medical history

He had no family history of ischemic heart disease or sudden cardiac death. He was a non-smoker and denied having a similar pain in the past or use of any drugs.

Differential diagnosis

Based on his symptoms, acute coronary syndrome, acute myocarditis, and aortic dissection were included in his differential diagnosis. 

Investigations

Laboratory investigations showed mild leukocytosis, along with elevated cardiac enzyme and homocysteine levels (Table 1). 

**Table 1:** Laboratory investigations ANA, antinuclear antibody; HDL; high-density lipoprotein; LDL, high-density lipoprotein

Parameters	Result	Normal Range
Complete Blood Count (CBC)		
Hemoglobin (Hb)	14 g/dL	12.0-15.0
Total Leukocyte Count (TLC)	14 × 10^9 ^/L	4.0-10.0
Platelets (Plt)	278 × 10^9 ^/L	150-400
Cardiac Enzymes		
Cardiac Troponin I	0.5	0-0.04 ng/mL
Creatine Kinase-MB (CK-MB)	232	5-25 IU/L
Creatine Phosphokinase (CPK)	2365	55-170 IU/L
Lactate Dehydrogenase (LDH)	557	140-280 IU/L
Homocysteine Levels	85	5-15 µmol/L
Liver Function Tests (LFTs)		
Albumin	4.5	3.4-5.4 g/dL
Bilirubin	0.6	0.1-1.2 mg/dL
Aspartate Aminotransferase (AST)	212	5-40 IU/L
Prothrombin Time (PT)	12	11-13.5 s
Activated Partial Thromboplastin Time (aPTT)	-	21-35 s
Renal Function Tests (RFTs)		
Urea	12	5-20 mg/dL
Creatinine	0.9	0.7-1.3 mg/dL
Lipid Profile		
Cholesterol	101	<200 mg/dL
Triglycerides	84	<150 mg/dL
HDL Cholesterol	62	>60 mg/dL
LDL Cholesterol	85	<100 mg/dL
Thyroid Stimulating Hormone (TSH)	3.5	0.4-4.5 mU/L
Protein C	Normal	-
Protein S	Normal	-
ANA	Negative	-

Polymerase chain reaction (PCR) for prothrombin gene mutation was negative. Cytogenetic analysis of the bone marrow showed no growth.

**Figure 2:**
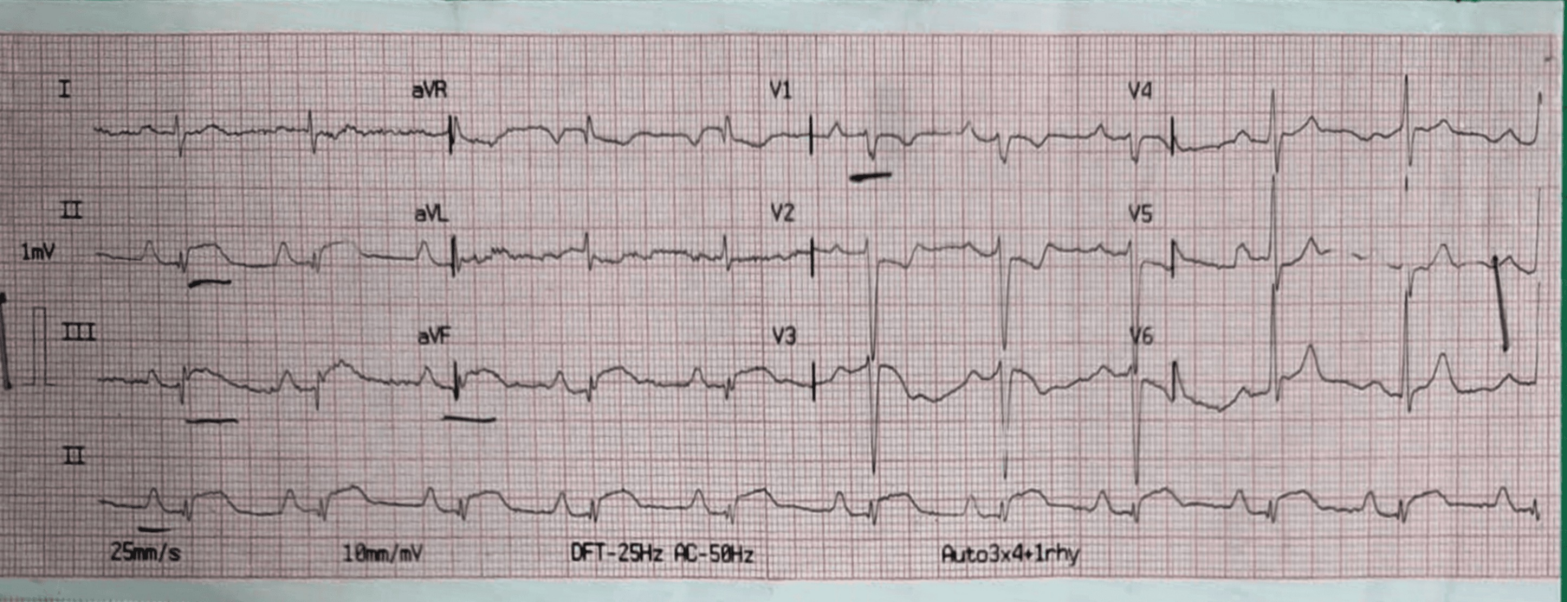
Electrocardiogram Prominent ST elevation in limb leads II, III, and augmented vector foot (aVF).

An electrocardiogram (ECG) showed ST elevation in limb leads II, III, and augmented vector foot (aVF) (Figure 2).

**Figure 3:**
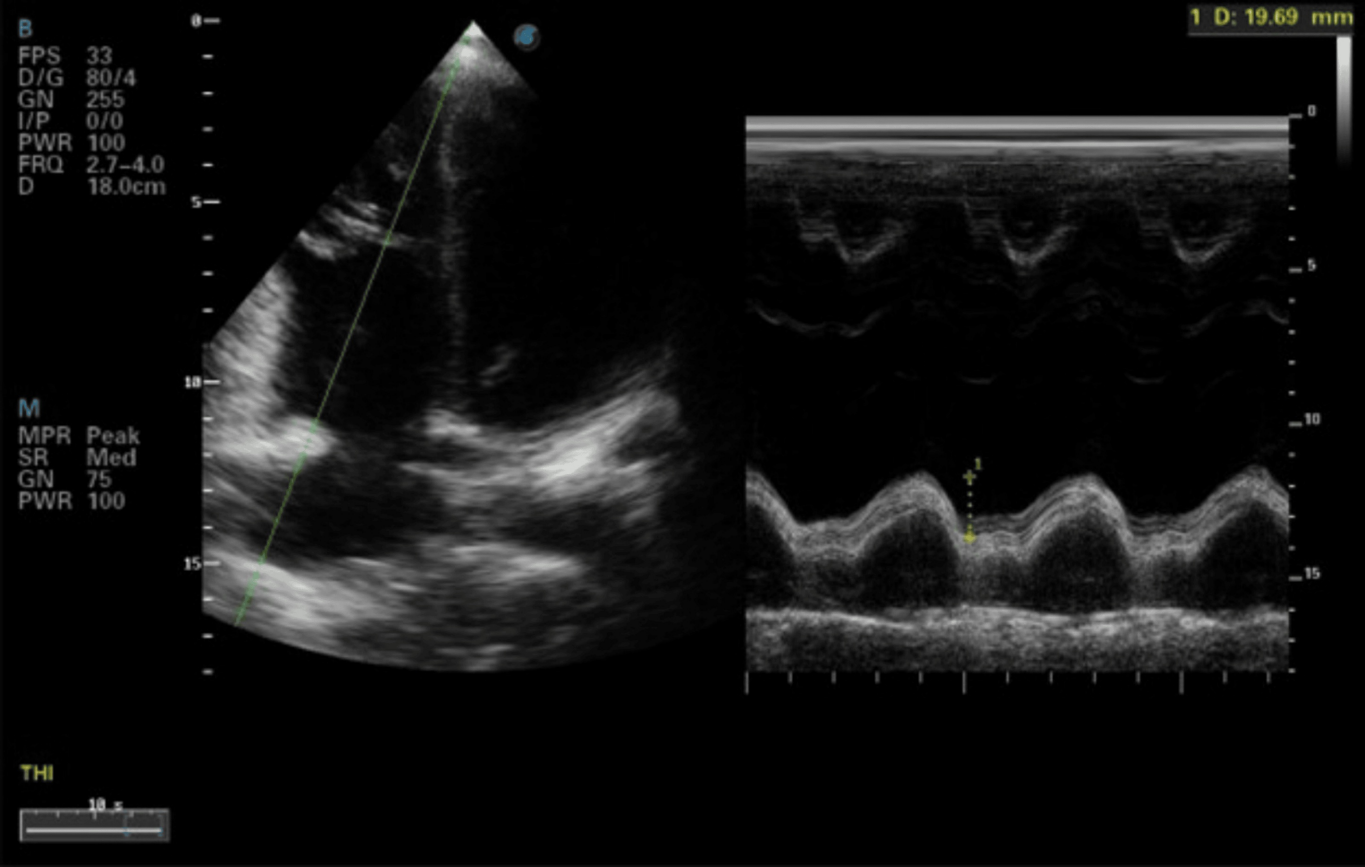
Echocardiography picture showing preserved RV systolic function with TAPSE of 19 mm RV, right ventricular; TAPSE, tricuspid annular plane systolic excursion

Echocardiography showed an ejection fraction (EF) of 45-50%, with inferio-posterior wall hypokinesia and jerk septal motion. Mid-to-distal right ventricular (RV) free wall hypokinesia with tricuspid annular plane systolic excursion (TAPSE) of 19 mm was observed (Figure 3). The right-sided chamber was prominent.

**Figure 4:**
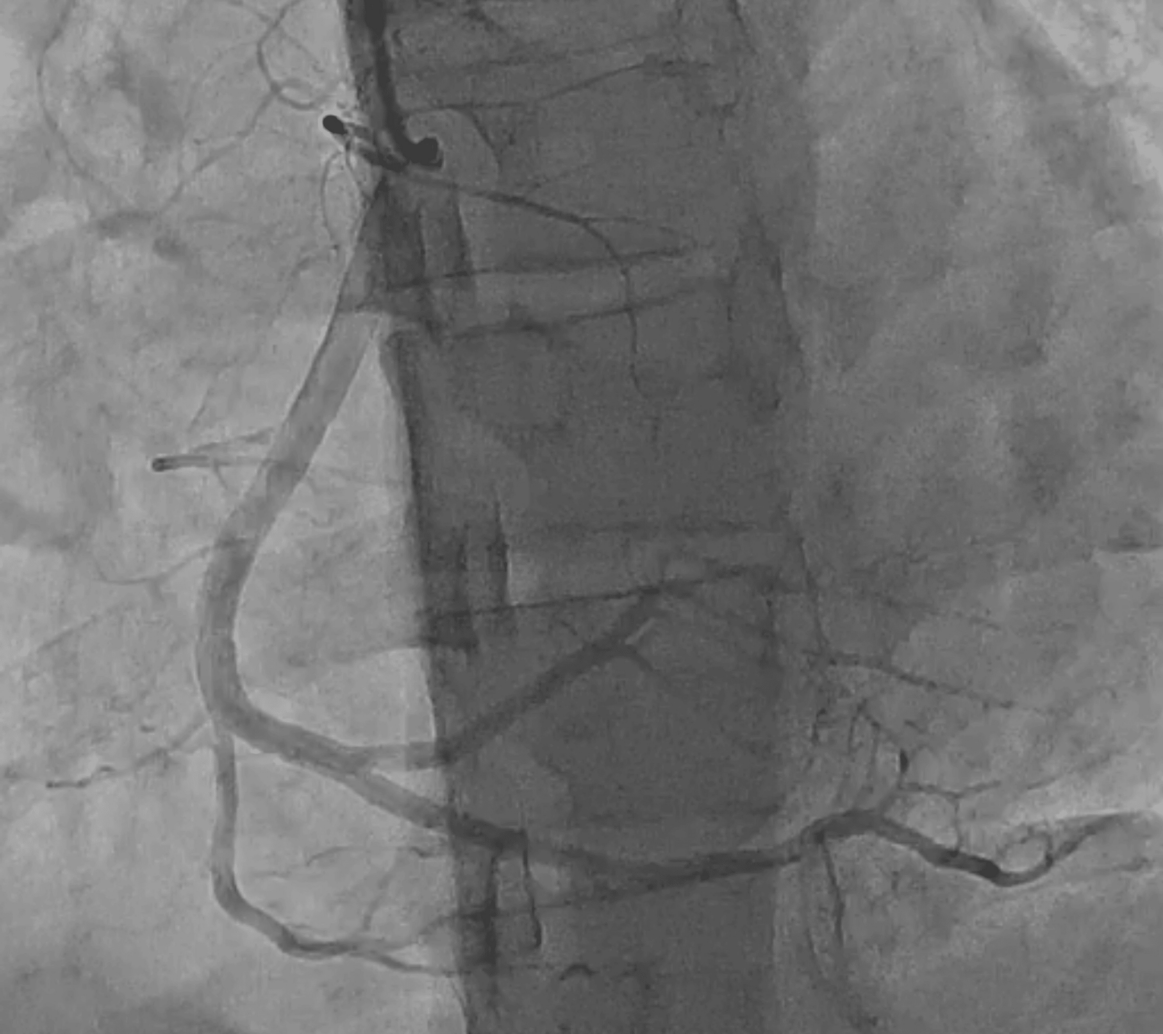
Coronary angiography Recanalized right coronary artery (RCA)

The patient was diagnosed with hyperhomocysteinemia (HHCY), which caused inferior wall MI. He was treated with thrombolysis using streptokinase. Later, angiography revealed a recanalized right coronary artery (RCA) with good thrombolysis in myocardial infarction (TIMI) III flow (Figure 4).

The patient was discharged on the fifth day with dual antiplatelet (aspirin 75 mg and clopidogrel 75 mg), anticoagulant (rivaroxaban 10 mg), beta-blocker (bisoprolol 2.5 mg), glyceryl trinitrate (2.6 mg), statin (rosuvastatin 20 mg), angiotensin-converting enzyme (ACE) inhibitor (enalapril 5 mg), folic acid, and multivitamins, with instructions to follow-up with a cardiologist within two weeks.

## Discussion

Homocysteine is a sulfur-containing amino acid whose normal concentration ranges from 5 to 15 µmol/L in the blood. However, when the levels exceed 15 µmol/L, it becomes HHCY [7]. HHCY is mainly caused by the dysfunction of enzymes and cofactors related to the biosynthesis of homocysteine. Other causes include vitamin B12, B6, and folate deficiency. Conditions like chronic renal insufficiency, hepatic dysfunction, Systemic Lupus Erythematosus, cancers, Marfan syndrome, hypothyroidism, and lifestyle factors, including smoking and alcohol abuse, can also lead to HHCY [8-9]. Traditional risk factors for MI include smoking, diabetes mellitus, hypertension, and hypercholesterolemia. Certain non-traditional risk factors are also known for MI in young adults, including HIV, systemic lupus erythematosus, obstructive sleep apnea, familial hypercholesterolemia, homocystinuria, antiphospholipid syndrome, and fibromuscular dysplasia [10].

Over the past few decades, extensive research has been conducted to elucidate the pathogenesis of MI associated with HHCY. Several studies have demonstrated that HHCY is a risk factor for cardiovascular diseases independent of classic risk factors such as smoking, hypercholesterolemia, diabetes mellitus, and hypertension [1]. The “arteriosclerosis” theory, previously expressed by McCully in 1969, supports the pathophysiological basis of association between HHCY and MI [11]. According to a meta-analysis conducted in 2008, every 5 µmol/L increase in homocysteine levels increased the risk of coronary heart disease by approximately 20% [12]. Similarly, another meta-analysis carried out in 2022 indicated that a reduction in homocysteine levels by 3 µmol/L reduced the risk of ischemic heart disease, stroke, and deep venous thrombosis occurrences by 16 %, 24%, and 25%, respectively [13].

In patients with acute coronary syndrome, HHCY is linked to thrombin generation and coagulation system activation. HHCY accelerates atherosclerosis by increasing vascular wall absorption of low-density lipoprotein (LDL) cholesterol and stimulating the growth of vascular smooth muscle [4]. HHCY may promote blood clot formation by increasing platelet aggregation and activation, leading to the formation of thrombi that block blood flow to the heart, causing MI. Elevated levels of homocysteine have also been shown to impair the production of nitric oxide, a molecule that helps maintain the normal functioning of the endothelium. This can lead to endothelial dysfunction, which is a precursor to the development of atherosclerosis and, ultimately, MI [14]. Thus, elevated total plasma homocysteine levels can cause vascular occlusion through thromboembolic events or endothelial dysfunction.

Treatment of HHCY depends on the underlying cause and severity of the condition. In cases where HHCY is caused by a deficiency in vitamin B12, folic acid, or vitamin B6, supplementation with these vitamins can effectively reduce homocysteine levels [15]. Lifestyle modifications, such as regular exercise, smoking cessation, and a healthy diet low in fat and cholesterol, can also help reduce the risk of cardiovascular disease associated with HHCY [16]. For patients with a history of MI, aggressive management of risk factors such as hypertension, diabetes, and dyslipidemia are crucial. This may involve the use of medications, such as ACE inhibitors or beta-blockers, to control blood pressure, cholesterol, and other risk factors [17]. Overall, the management of HHCY requires a multifaceted approach that addresses both the underlying cause of the condition and the associated risk factors for cardiovascular disease.

Follow-up

The patient attended the cardiology outpatient department after two weeks, and his symptoms have been under control since then. He is taking his medications regularly and is doing well.

Learning objectives

1. This case report helps to recognize HHCY as an independent risk factor for MI, especially in young patients who do not have traditional risk factors.

2. It also emphasizes the need to apply appropriate diagnostic and management strategies for MI caused by HHCY.

3. It focuses on how to timely integrate a multidisciplinary approach for managing patients with marfanoid phenotypes and elevated homocysteine levels to prevent cardiovascular complications.

## Conclusions

This case emphasizes the importance of recognizing HHCY as a potential risk factor for MI, especially in young individuals. Early diagnosis and management of HHCY are crucial to prevent cardiovascular complications. Aggressive management of the risk factors for cardiovascular diseases is important for preventing further events.
